# 590 Resuscitation with Enteral Fluids: A Prospective Observational Study to Reduce IV-related Edema (REFORM)

**DOI:** 10.1093/jbcr/iraf019.219

**Published:** 2025-04-01

**Authors:** Amanda Wiggins, Leopoldo Cancio

**Affiliations:** United States Army Institute of Surgical Research Burn Center; United States Army Institute of Surgical Research Burn Center

## Abstract

**Introduction:**

Enteral resuscitation (ER) may be particularly useful in resource-limited areas and on the battlefield. The Resuscitation with Enteral Fluids: a prospective observational study to reduce IV-related edema (REFORM) aimed to evaluate ER in burn patients with < 60% total body surface area (TBSA) burns.

**Methods:**

This was a single-site, non-randomized, prospective observational study of ER in patients admitted to the burn ICU. The World Health Organization oral rehydration salts (ORS) were administered through a nasogastric or orogastric tube (NGT, OGT). Patients were excluded from receiving ORS if they had any of the following: GI tract not appropriate for enteral fluid resuscitation (e.g., recent gastric bypass surgery or GI tract not in continuity); significant vasopressor use, i.e. norepinephrine > 0.15 mcg/kg/min (with or without vasopressin); burn size ≥ 60% TBSA; admission ≥ 24 hours postburn; age ≥ 70 years; or high-voltage electric injury or chemical injury.

**Results:**

A total of 8 patients received ORS. The median age was 47 years (IQR 44.5-52); median burn size 30.75% TBSA (IQR 23.5-37.4); 75% male.

The median total fluid delivered during the first 24 hours postburn was 4.9 ml/kg/%TBSA (IQR 4.4-5.4) or 144.6 ml/kg (IQR 115.8-187.7). Median intravenous (IV) crystalloid fluids were 2.7 ml/kg/%TBSA (IQR 2-3.5); median colloid fluids were 0.1 mL/kg/%TBSA (IQR 0-0.3); median enteral fluid delivered was 1.7 ml/kg/%TBSA (IQR 1-2.8), 56 mL/kg (IQR 22-70), and median total volume of 4242 mL (IQR 2174-5705).

All patients survived to 28 days after admission. One patient ultimately died of complications related to right heart failure from pulmonary hypertension, unrelated to his burn resuscitation. Median length of hospital stay was 56 days (IQR 45.8-66.8), and median ICU length of stay was 37.5 days (IQR 19-50.8). Most patients did not require vasopressor support (63%) during resuscitation, and none required emergent exploratory laparotomy. Most patients (75%) did not develop a ventilator-associated pneumonia during their first week of admission.

**Conclusions:**

ER enabled a reduction in IV fluid requirements to a level between the Brooke and Parkland estimations (median of 2.7 ml/kg/%TBSA). This indicates that enteral resuscitation is effective at providing a large proportion of total fluid resuscitation needs.

**Applicability of Research to Practice:**

These results suggest that ORS can be safely administered to burn patients with less than 60% TBSA. ORS offers advantages over IV therapy in austere or resource limited settings; it requires only small packets of glucose and electrolytes and a source of clean water.

**Funding for the Study:**

This study was funded by the department of defense.

